# Left ventricular synchrony, torsion, and recoil mechanics in Ebstein’s anomaly: insights from cardiovascular magnetic resonance

**DOI:** 10.1186/s12968-017-0414-y

**Published:** 2017-12-14

**Authors:** Michael Steinmetz, Simon Usenbenz, Johannes Tammo Kowallick, Olga Hösch, Wieland Staab, Torben Lange, Shelby Kutty, Joachim Lotz, Gerd Hasenfuß, Thomas Paul, Andreas Schuster

**Affiliations:** 1 Department of Pediatric Cardiology and Intensive Care Medicine, Georg-August University Göttingen, University Medical Center, Robert-Koch-Str. 40, D-37099 Göttingen, Germany; 2 Institute for Diagostic and Interventional Radiology, Georg-August-University Göttingen, University Medical Center, Göttingen, Germany; 3 Department of Cardiology and Pneumology, Georg-August-University Göttingen, University Medical Center, Robert-Koch-Str. 40, D-37099 Götttingen, Germany; 4 DZHK (German Centre for Cardiovascular Research), partner site Göttingen, Göttingen, Germany; 5 University of Nebraska Medical Center/ Children’s Hospital and Medical Center, Omaha, NE USA; 6 Department of Cardiology, Royal North Shore Hospital, The Kolling Institute, Nothern Clinical School, University of Sydney, Sydney, Australia

**Keywords:** Ebstein anomaly, CMR feature tracking, Left ventricle, Dyssynchrony, Torsion and recoil, Heart failure, Congenital heart disease

## Abstract

**Background:**

Disease progression and heart failure development in Ebstein’s Anomaly (EA) of the tricuspid valve is characterized by both right and left ventricular (LV) deterioration. The mechanisms underlying LV dysfunction and their role in heart failure development are incompletely understood. We hypothesized that LV dyssynchrony and impaired torsion and recoil mechanics induced by paradoxical movement of the basal septum may play a role in heart failure development.

**Methods:**

31 EA patients and 31 matched controls underwent prospective cardiovascular magnetic resonance (CMR). CMR feature tracking (CMR-FT) was performed on apical, midventricular and basal short-axis and 4D–volume analysis was performed using three long-axis views and a short axis cine stack employing dedicated software. Circumferential uniformity ratio estimates (CURE) time-to-peak-based circumferential systolic dyssynchrony index (C-SDI), 4D volume analysis derived SDI (4D–SDI), torsion (Tor) and systolic (sysTR) and diastolic torsion rate (diasTR) were calculated for the LV. QRS duration, brain natriuretic peptide, NYHA and Total R/L-Volume Index (R/L Index) were obtained.

**Results:**

EA patients (31.5 years; controls 31.4 years) had significantly longer QRS duration (123.35 ms ± 26.36 vs. 97.33 ms ± 11.89 *p* < 0.01) and showed more LV dyssynchrony (4D–SDI 7.60% ± 4.58 vs. 2.54% ± 0.62, *p* < 0.001; CURE 0.77 ± 0.05 vs. 0.86 ± 0.03, p < 0.001; C-SDI 7.70 ± 3.38 vs. 3.80 ± 0.91, *p* = 0.001). There were significant associations of LV dyssynchrony with heart failure parameters and QRS duration. Although torsion and recoil mechanics did not differ significantly (*p* > 0.05) there was an association of torsion and recoil mechanics with dyssynchrony parameters CURE (sysTR *r* = −0.426; *p* = 0.017, diasTR *r* = 0.419; *p* = 0.019), 4D–SDI (sysTR *r* = 0.383; *p* = 0.044) and C-SDI (diasTR *r* = −0.364; *p* = 0.044).

**Conclusions:**

EA is characterized by LV intra-ventricular dyssynchrony, which is associated with heart failure and disease severity parameters. Markers of dyssynchrony can easily be quantified from CMR-FT, and may have a role in the assessment of altered cardiac function, carrying potential management implications for EA patients.

**Electronic supplementary material:**

The online version of this article (10.1186/s12968-017-0414-y) contains supplementary material, which is available to authorized users.

## Background

Ebstein’s anomaly (EA) of the tricuspid valve is a rare but clinically important heart defect that accounts for approximately 0.5% of congenital cardiac malformations and occurs in about 1 per 200, 000 live births [1]. The displacement of the dysplastic and tethered tricuspid valve creates “atrialisation” of the right ventricle (RV), leading to right atrial overload, significant tricuspid valve regurgitation and obstruction of the right ventricular outflow tract [1, 2].

Although EA is primarily a RV disease it also affects the shape and function of the left ventricle (LV). Paradoxical movement of the basal interventricular septum and LV dysfunction have been described [3]. Dyssynchrony and impaired ventricular contraction and relaxation may be manifestations of disturbances in myocardial function [4].

Due to advances in heart failure therapy through pacemaker based cardiac resynchronization therapy, the assessment of cardiac dyssynchrony based on imaging has gained increasing attention [5, 6]. Dyssynchrony can be quantified from electrocardiograms (ECG), echocardiography and cardiovascular magnetic resonance imaging (CMR) [7]. Several methods to assess dyssynchrony using CMR balanced steady state in free precession (bSSFP) images are available [4, 7–9]. Using CMR based feature tracking (FT), a relatively new CMR based technique, several dyssynchrony parameters including circumferential uniformity ratio estimate (CURE) and time-to-peak-based circumferential systolic dyssynchrony index (C-SDI) can be obtained. Torsion and recoil mechanics, which describe LV twisting and untwisting motion can also be obtained [9]. Non-FT based dyssynchrony parameters such as the 4D–SDI can also be measured [4, 10, 11]. In contrast to right heart failure, the mechanisms underlying LV dysfunction, and their role in heart failure development are incompletely understood in EA. We hypothesized that LV dyssynchrony and altered torsion and recoil mechanics induced by paradoxical movement of the basal septum may play a role in heart failure development. We consequently analyzed these parameters using comprehensive CMR-FT based deformation imaging in a unique cohort of EA patients.

## Methods

### Study population

The database of the Department of Pediatric and Adult Congenital Heart Disease at the Georg-August-University Medical Center Göttingen, Germany, holds records of 58 patients with EA. After using exclusion criteria like CMR incompatible metallic implants, claustrophobia, age < 10 years and additional severe congenital heart malformations, 31 (20 male) patients were recruited for the study. CMR and all other exams were performed prospectively within one day from 02/2013 until 04/2015.

Previous surgical treatment of EA was present in six patients (*n* = 1 tricuspid valve replacement, *n* = 2 Glenn-Anastomosis and *n* = 3 tricuspid valve reconstructions). Seven patients had an atrial septal defect, which had already been closed by surgery or catheter intervention in six cases.

Medical history, clinical examination, laboratory tests including heart failure parameters like brain natriuretic peptide (BNP), ECG-derived measures (QRS and QTc-duration), New York Heart Association (NYHA) functional class and CMR were part of the study protocol. An age- matched group of 31 healthy subjects served as a control group.

### CMR imaging

The study was performed employing current CMR guidelines for patients with congenital heart disease using a 1.5 Tesla CMR scanner (Symphony, Siemens Healthineers, Erlangen, Germany) [12]. All patients were examined without sedation according to a standardized imaging protocol for EA.

Ventricular dimensions and function were assessed using stacks of multislice-multiphase bSSFP cine images in ventricular short-axis and three long axis views. Standard imaging parameters included: repetition time (TR) =14 ms, echo time (TE) =2.6 ms, flip angle = 20°, slice thickness = 5 mm, spatial resolution 1.3 × 2.5 mm; parallel imaging acceleration factor 2.

All images were obtained during breath holding based on retrospective ECG-gating to minimize artifacts. Both volumetric analysis of all chambers and calculation of the Total Right-Left-Volume-Index as a severity parameter of EA were performed using commercially available software (QMass, Medis BV, Leiden, The Netherlands) as described previously [13].

### Feature tracking

Myocardial feature tracking was performed using dedicated software (2D CPA MR, Cardiac Performance Analysis, Version 1.1.2.36, TomTec Imaging Systems, Unterschleissheim, Germany).

From the short axis stack three planes were identified and analyzed: one basal plane defined as the last slice exhibiting circular myocardium throughout the entire cardiac cycle, an apical plane that still showed an LV cavity during systole and the plane in between the two planes defined as midventricular slice.

LV endo- and epicardial borders were manually drawn in all three planes using a point-and-click approach in LV end-diastole. The FT algorithm automatically tracks 48 points based on anatomic elements along the cavity-myocardial tissue border throughout the cardiac cycle. In case of insufficient semi-automatic border tracking, the initial contours were manually corrected and the FT algorithm reapplied. From the FT analysis, strain as a measure of the magnitude of myocardial contraction and relaxation [4], as well as the time until the strain maximum is reached (time-to-peak, TPK) can be obtained in different orientations of the heart.

The FT software used creates strain and TPK measures of the 48 tissue points itself as well as segmental data using the 16-segment-modell of the American Heart Association.

All results reported in the current paper are based on averaging of three repeated measurements and analyses in order to maximize reproducibility as previously demonstrated [14].

### Dyssynchrony analysis

#### TPK based systolic Dyssynchrony index

**Fig. 1:**
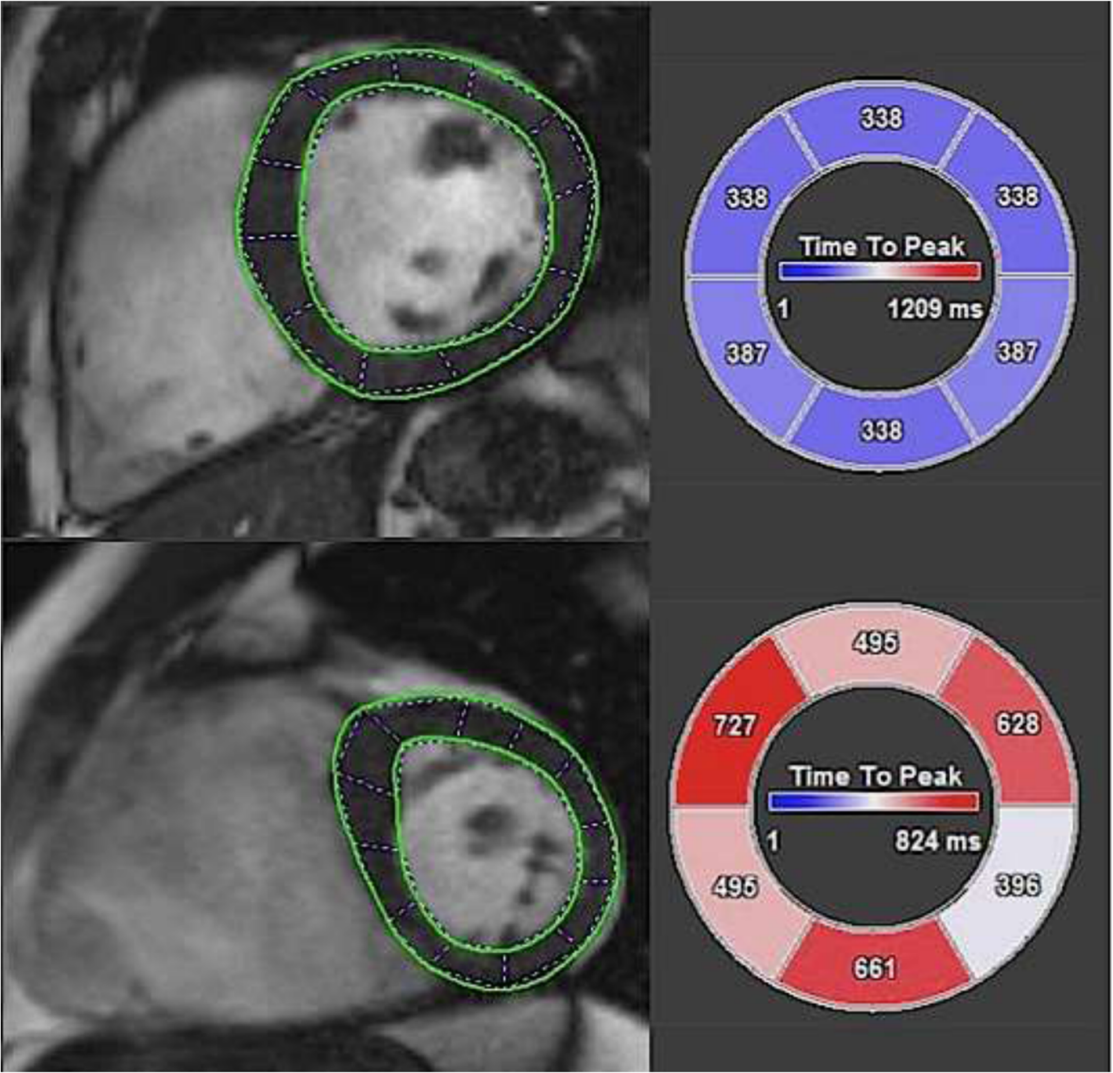
Comparison of TPK-values from basal segments showing almost equal TPK values in the control (top) and varying TPK measures in an EA patient (bottom)

Using FT analysis circumferential strain and TPK measures can be obtained for each cardiac segment (Fig. 1). For each segmental TPK-value its percentage of the duration of the cardiac cycle ($\frac{TPK\  Segment}{\mathrm{RR}-\mathrm{intervall}}\Big)$ was calculated. A Systolic Dyssynchrony Index (SDI) defined as the standard deviation (SD) of the calculated TPK-percentages of all segments was established. The circumferential SDI (C-SDI) was calculated as follows: $- SDI= SD\Big(\frac{TPK\  Segment\ 1}{RR- Intervall};\frac{TPK\  Segment\ 2}{RR- Intervall};\dots; \frac{TPK\  Segment\ 16}{RR- Intervall}$).

#### Uniformity ratio estimate

**Fig. 2:**
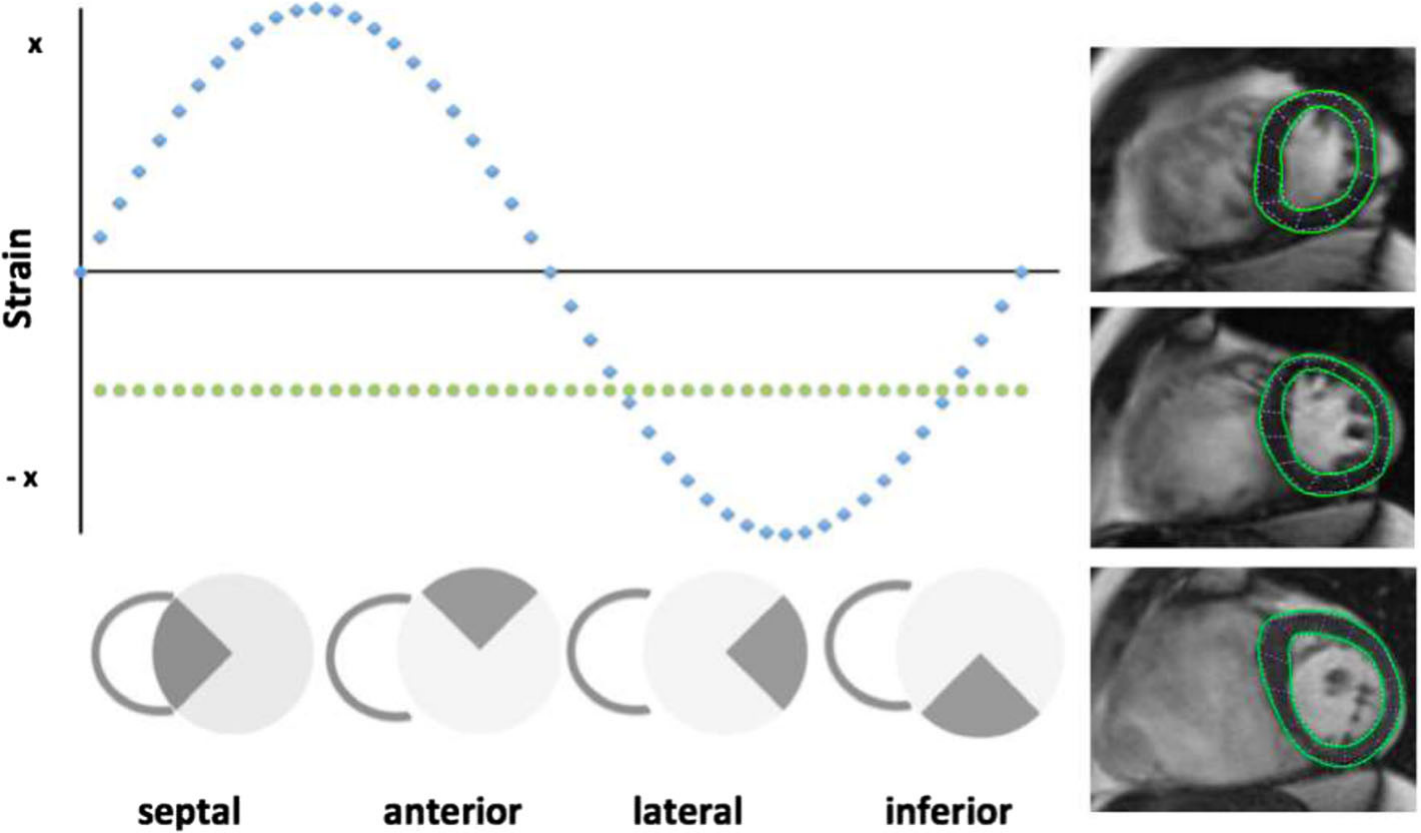
Comparison of different strains (at a given time point) with spatial position. The 48 hypothetical points show: 1. Complete dyssynchrony (blue dots) where opposite segments exhibit different strain 2. Perfect synchrony (green points) all segments have identical Strain. Modified from Taylor et al. [4]. SSSFP short axis slices with corresponding feature tracking segmentation in an EA patient, from top to bottom: apical, midventricular and basal slice

Using FT based strain analysis of the 48 tissue voxels, uniformity ratios of the LV can be calculated. Circumferential Uniformity Ratio Estimate (CURE) represents the spatial uniformity of strain over all time points and across all slices. Assuming that in a completely dyssynchronous heart opposing walls develop opposing strain directions, a perfect synchronous heart will exhibit similar strain directions in all segments at a given point in time which can be expressed by the CURE [4] (Fig. 2).

CURE was calculated as previously described [4]: *CURE* = (*A* ^2^∫_0_[*A* ^2^_0_ + 2*A* ^2^])^1/2^, where A [2] o and A^2^1 are the spatial and temporal sum of the zero and first order power terms, respectively. Measures range from CURE = 1 representing perfect synchrony and CURE = 0 for complete dyssynchrony [15].

#### 4D–LV-analysis

**Fig. 3:**
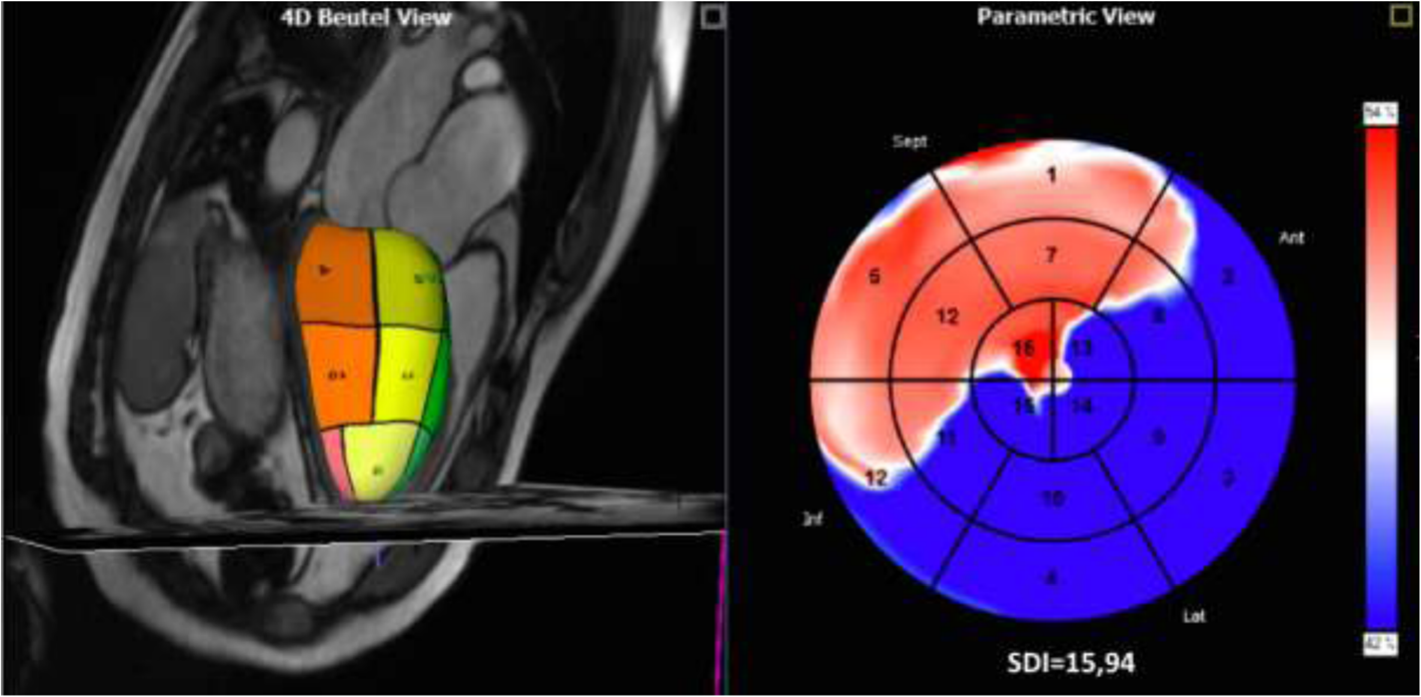
Left: 3D–model of the LV of a patient with EA with segmental subdivision in a three chamber view; Right: Map of myocardial activation representing a delay of the septal segments (blue = activated; red = not yet actived)

The prototype software 4D–LV-Analysis (TomTec Imaging Systems) uses three long-axis views and a short axis stack (1 short axis) to create a 4D representation of the LV volume changes during a cardiac cycle. End-diastolic and end-systolic endocardial borders were manually drawn in 2-, 3-, and 4-chamber views and automatically propagated onto the short axis views. Based on the outlined contours, a 4D model of LV contraction over one cardiac cycle was generated and automatically subdivided into 16 segments according to a modified 16-segment American Heart Association bulls-eye plot (Fig. 3). For each segment time-volume curves were created and used to generate a map that illustrates the dispersion mechanism of LV contraction. A 4D–SDI, defined as the standard deviation of time to reach minimum regional volume for all 16 segments, was calculated.

### Torsion and recoil

Torsion and torsion rates were calculated as described using Excel 2011 for Macintosh (Microsoft Corporation, Redmond, Washington, USA) [16]. Basal clockwise rotation (∅*basal*) and apical counter-clockwise rotation ( ∅ *apical*) were obtained using rotational displacement data of all 48 tissue points in corresponding slices. The distance (*D*) between basal and apical planes was calculated considering slice thickness and inter-slice gap. Torsion was defined as the difference of ∅*basal* and ∅ *apical* normalized to LV length: $Torsion=\frac{\ \left(\varnothing apical\right)-\left(\varnothing basal\right)}{(D).}$.

Global LV Torsion was calculated averaging subepicardial and subendocardial torsion. Peak systolic torsion rate (sysTR) and peak diastolic torsion rate (diasTR) were generated using the first derivative of torsion.

### Statistical analysis

Data are expressed as mean ± standard deviation. The Shapiro-Wilk-Test was used to test for normal distribution of the data. Since there was no normal distribution, data of EA and control group were compared using the Mann-Whitney-U test for non-parametric data. To represent potential relations between clinical parameters and obtained dyssynchrony- and torsion measures, Spearman’s correlation coefficients were calculated. All statistical tests with *p* values <0.05 were considered statistically significant. For inter-observer variability, two independent observers (SU & TL) analyzed long and short axis stacks with CMR-FT and 4D–LV-analysis of 10 randomly selected patients with EA and 10 controls. Intra-observer variability was calculated after reanalysis of 10 randomly selected patients and 10 controls by the first observer (SU) six weeks after primary analysis. For both inter- and intraoberver variability the following parameters were assessed: 1. Intraclass Correlation Coefficents (ICC) 2. Coefficients of Variation (CoV) and 3. Bland-Altman-Analysis. Statistical analysis was performed using Microsoft Excel (Microsoft Corporation) and SPSS Statistics (v 22, International Business Machines, Armonk, New York, USA).

## Results

CMR-FT was successfully performed in all 31 patients and controls. The 4D–LV-Analysis was successfully performed in 28 patients and 31 controls. Three patients had to be excluded due to image artifacts and inability of the prototype software to create the 4 D volume curves. Patient characteristics can be found in Table 1.

**Table 1:** Comparison of EA patients and controls

	Patients with EA	Controls	*P*-value
Age [years]	31.6 ± 16,9	31.4 ± 16.5	0.972
male/female	19/12	17/14	0.610
ECG			
QRS-duration [ms]	*123.35 ± 26.36*	*97.33 ± 11.89*	< 0.001
QTc-duration [ms]	*424.26 ± 37.19*	*400.67 ± 23.66*	0.004
Heart failure			
NYHA	2.62 ± 0.62	n/A	
BNP [ng/l]	78.32 ± 128.85	n/A	
Disease severity			
R/L Index	2.58 ± 1.66		
Dyssynchrony,			
C-SDI [%]	*7.70 ± 3.38*	*3.80 ± 0.91*	< 0.001
CURE	*0.77 ± 0.05*	*0.86 ± 0.03*	< 0.001
4D–SDI [%]	*7.60 ± 4.58*	2.54 ± 0.62	< 0.001
LV Volumetric Paramters			
EDVi [ml/m2]	*72.41 ± 11.13*	*82.67 ± 15.14*	0.012
ESVi [ml/m2]	29.78 ± 8.66	27.55 ± 8.02	0.135
EF [%]	*58.82 ± 7.94*	*67.03 ± 5.41*	< 0.001

Significant differences (*p* < 0.05) are printed italic

### Left ventricular dyssynchrony

Patients with EA showed significantly higher LV dyssynchrony in all CMR derived parameters compared to controls (Table 1).

In EA patients, dyssynchrony parameters C-SDI and CURE showed significant correlations with heart failure parameters NYHA and BNP, as well as the electrical dyssynchrony markers QRS and QTc as measures of depolarisation and repolarisation (Table 2). LV 4D–SDI showed significant correlation with both NYHA and ECG-data as well as a trend towards correlation with BNP (Table 2).

**Table 2:** correlation of CMR derived measures of dyssynchrony (C-SDI, CURE, 4D–SDI) with established heart failure markers and ECG markers of dyssynchrony, as well as the R/L Index as a marker of EA severity

Indices	C-SDI	CURE	4D–SDI
Clinical Data			
NYHA	*r* =* 0.529*; *p* =* 0.004*	*r* = *−0.429*; *p* = *0.023*	*r* = *0.496*; *p* = *0.018*
BNP	*r* = *0.436*; *p* = *0.018*	*r* = *−0.508*; *p* =* 0.005*	*r* = 0.363; *p* = 0.068
QRS-duration	*r* =* 0.460*; *p* = *0.009*	*r* = *−0.495*; *p* = *0.005*	*r* = *0.551*; *p* = *0.002*
QTc-duration	*r* = *0.378*; *p* = *0.036*	*r* = *−0.371*; *p* = *0.040*	*r* = *0.569*; *p* = *0.002*
Severity Classification			
R/L - Index	*r* =* 0.419*; *p* = *0.019*	*r* = *−0.474*; *p* = *0,007*	*r* = *0.502*; *p* = *0.007*

Significant correlations (*p* < 0.05) are printed italic

Moreover, we detected correlations of fRV ejection fraction (EF) with dyssynchrony markers 4D–SDI and C-SDI (Additional file 1: Table S4).

### Torsion and recoil mechanics

Compared to the control group, patients with EA did not exhibit any significant difference of neither global Tor, sysTR nor diasTR (Table 3). However, basal rotation in EA was significantly increased compared to controls (Table 3).

**Table 3:** Comparison of torision and recoil mechanics of EA patients and controls

	Patients with EA	Controls	*p*-value
Basal rotation (°)	5.79 ± 4.57	4.07 ± 2.24	0.048
Apical rotation (°)	5.45 ± 2.71	4.41 ± 2.42	0.119
Torsion [°/cm]	1.64 ± 1.18	1.45 ± 1.00	0.519
SystolicTorsion Rate [°/cm/s]	12.23 ± 6.22	10.40 ± 5.42	0.123
Diastolic Torsion Rate [°/cm/s]	−10.67 ± 6.88	−12.47 ± 6.61	0.127

Whilst all torsion and recoil data showed a significant correlation with the LV dyssynchrony measure CURE, only sysTR and diasTR, respectively, showed correlation with C-SDI and 4D–SDI (Table 4). Moreover, torsion exhibited significant correlation with LV EF (*r* = 0,459; *p* = 0,009)

**Table 4:** Correlation of torsion and recoil parameters with CMR parameters of dyssynchrony, heart failure, ECG and EA severity

	Torsion	syst. Torsion Rate	diast. Torsion Rate
Dyssynchrony measures			
C-SDI	*r* = 0.082; *p* = 0.660	*r* = −0.106; *p* = 0.569	*r* = *−0.364*; *p* = *0.044*
CURE	*r* = *−0.355*; *p* =* 0.049*	*r* =* −0.426*; *p* = *0.017*	*r *= *0.419*; *p* = *0.019*
4D–SDI	*r* = 0.123; *p* = 0.532	*r* = *0.383*; *p* = *0.044*	*r* = −0.256; *p* = 0.173
Clinical data			
NYHA	*r* = 0.194; *p* = 0.322	*r* = 0.174; *p* = 0.376	*r* = −0.126; *p* = 0.524
BNP	*r* = 0.273; *p* = 0.151	*r* = *0.391*; *p* = *0.036*	*r* = −0.337; *p* = 0.074
QRS-duration	*r* = 0.191; *p* = 0.304	*r* = 0.196; *p* = 0.289	*r* = −0.228; *p* = 0.218
Severity index			
R/L-Index	*r* = −0.052; *p* = 0.780	*r* = 0.094; *p* = 0.616	*r* = 0.038; *p* = 0.837

Significant correlations (*p* < 0.05) are printed italic

In terms of heart failure and EA severity, there was a significant correlation of sysTR only with BNP (Table 4).

### Subgroup analysis of EA patients after surgery/ with ASD

We intentionally included EA patients who received previous right heart surgery or atrial septal defect closure since we focused on the LV and aimed to analyze LV dyssynchrony in the whole spectrum of EA. After surgery, EA patients often keep their “visual dyssynchrony” on imaging studies. A subgroup analysis of the operated EAs and the remaining atrial septal defect patient revealed similar results as for the complete cohort with regard to dyssynchrony and torsion/recoil mechanics (data supplied in Additional file 1: Table S1and S2.).

### Reproducibility

Parameters could be reproduced with sufficient accuracy on an inter- and intraobserver level (see Tables 5 and 6 and Additional file 1: Table S3).

**Table 5:** Intraobserver variability of CMR segmentation for dyssynchrony and torsion/ recoil markers for 10 randomly selected selected 10 controls and 10 EA EA patients

	ICC	CoV	Bland-Altmann-analysis
4D–SDI	0.99	8.39	0.60 (−0.51–1.71)
R-SDI	0.89	24.58	2.66 (−2.05–7.37)
C-SDI	0.94	20.42	1.34 (−1.98–4.67)
L-SDI	0.97	14.02	1.48 (−1.04–3.99)
RURE	0.94	4.22	0.05 (−0.02–0.12)
CURE	0.96	2.50	0.03 (−0.01–0.05)
Torsion	0.93	26.83	0.30 (−0.31–0.91)
Systolic Torsion Rate	0.90	15.66	1.85 (−1.02–4.72)
Diastolic Torsion Rate	0.90	17.43	2.25 (−1.66–6.69)

**Table 6:** Interobserver analysis of CMR segmentation for dyssynchrony and torsion/ recoil markers for 10 randomly selected 10 controls and 10 EA patients

	ICC	CoV	Bland-Altmann-analysis
4D–SDI	0.91	17.12	0.94 (−0.65–2.53)
R-SDI	0.95	12.97	1.67 (−0.66–4.01)
C-SDI	0.95	14.91	1.08 (−0.77–2.93)
L-SDI	0.98	12.62	1.03 (−0.68–2.73)
RURE	0.88	5.24	0.04 (−0.03–0.12)
CURE	0.97	2.73	0.02 (−0.01–0.04)
Torsion	0.89	30.98	0.42 (−0.50–1.34)
Systolic Torsion Rate	0.83	19.12	2.89 (−1.53–7.30)
Diastolic Torsion Rate	0.88	19.08	2.45 (−1.88–6.79)

## Discussion

The main finding of this investigation is that EA patients exhibit intra LV dyssynchrony, which can be measured from routine CMR cine SSFP images using CMR FT and 4D analysis. The dyssynchrony may be in part the mechanism behind LV dysfunction and overall cardiac deterioration in EA, and may be of potential value in surgical decision-making. CMR-derived dyssynchrony measures CURE, C-SDI and 4D–SDI showed a higher degree of intra LV dyssynchrony in our cohort of EA compared to healthy controls. These parameters correlated well with the established global ECG based electrical dyssynchrony markers, QRS and QTc. This supports the assumption that CMR actually measures the “visual” equivalent of ECG changes that translate into myocardial abnormalities and dyssynchrony, i.e. deterioration of function. Interestingly, EA patients with signs of heart failure (as expressed by higher NYHA functional class or BNP values) or a more severe form of EA (as expressed by the R/L-Volume index) exhibited more intra-LV dyssynchrony.

The deleterious effect of a malfunctioning or malformed RV on the LV has been described for various congenital cardiac malformations affecting primarily the right heart [17], including tetralogy of Fallot [18], atrial septal defect [19] and EA [13]. In a recent study, we have demonstrated that measures of right sided atrial and ventricular deformation (strain and strain rate) are altered in EA and are associated with heart failure markers [20]. Even though EA is primarily a right heart malformation, LV function is also altered in EA. However, the mechanism behind LV deteroration in EA remains elusive. The basal LV septal dyssynchrony in EA observed by echocardiography or CMR 4CV has often been described to play a role [3]. 

The indication and timing for surgery in the form of tricuspid valve repair or replacement is still a matter of debate in EA [21]. Some centers advocate early surgical correction using tricuspid valve reconstruction [22]. Other centers do not generally operate EA patients, except those with a disease severity requiring immediate postnatal surgery. This is because the natural history of EA suggests a favorable course without tricuspid valve reconstruction [23, 24]: Patients with even moderate to severe EA can reach adulthood and do not often exhibit signs of heart failure until they reach 3rd or 4th decade of life [25]. Due to the lack of high numbers of EA patients cared for in any individual center, and a lack of multicenter studies, there is little evidence and objective criteria with regard to the optimal timing for surgery (tricuspid valve reconstruction or Glenn). Personal and single center experiences drive real-life management in most cases.

Findings of this study suggest that the LV dyssynchrony described here may be a sign of impaired LV function in EA patients. Due to the relatively small sample size, we are unable to calculate cut-off values for measures of dyssynchrony (SDI, CURE), thus alerting the clinician to evaluate for tricuspid valve reconstruction, replacement or 1 and ½ repair (Glenn) in an individual patient. However, in combination with exercise testing and the total R/L index, the signs of LV dyssynchrony identified in this investigation could be a valuable tool in the detection of EA patients at risk for heart failure. This notion is underpinned by the correlation of dyssynchrony parameters with heart failure markers BNP and NYHA class observed in our study. Assenza’s observation that a fragmented and prolonged QRS complex (the ECG equivalent of dyssynchrony) identifies patients with a more severe EA including more pronounced functional impairment and worse clinical profile [26], is keeping in with our findings relating dyssynchrony from imaging (C-SDI, 4D–SDI, CURE), heart failure markers (BNP, NYHA class) and severity of EA (R/L-Index). Moreover, the correlation of 4D SDI and C-SDI with fRV EF is a hint that RV function also impacts on LV dyssynchrony.

It is noteworthy that torsion and recoil mechanics appear to be preserved in EA, while they are frequently reduced in classic systolic heart failure conditions [16, 27, 28]. In our cohort, the correlation of sysTR with heart failure markers was limited to BNP, global torsion was preserved, but basal rotation was increased in EA. Thus, we can only speculate that the normal LV torsion and recoil values observed in our cohort do not represent preservation but rather a compensatory upregulation of torsion and recoil mechanics. We hypothesize that EA can be associated with a compensatory torsion increase. This is backed by tissue Doppler echocardiographic studies by Park et al. [29] (decreased torsion in mild, but compensatory normalization in moderate to severe diastolic heart failure), Cameli et al. [30] (“hypertorsion” in early stages of hypertensive heart disease vs. normalization and decrease in late severe hypertensive heart disease) and a CMR study by Ruessel et al. [31] (increased LV torsion in hypertrophic cardiomyopathy gene mutation carriers). The poor correlation of torsion and recoil with heart failure markers and disease severity may be due to the low number of EA patients with higher degree heart failure and severe EA degrees in our cohort.

### Limitations

This is a cross sectional study with a relatively small sample size and thus we cannot comment on changes in dyssynchrony as well as torsion/recoil markers and their prognostic impact over time. Correlation of synchrony with the other parameters studied is modest. Statistical significance may be impaired by small patient numbers. Due to the small sample size, multiple comparisons adjustments were not carried out, which is a statistical limitation of this study. However, the number of EA patients included is comparable with those reported in other published studies and due to the rarity of the disease inclusion of higher patient numbers is very difficult. A longitudinal and preferably multicentric study with more patients would be required to look at prognostic impact and possibly obtain higher statistical validity and correlation values. However, the present study can serve as a proof of principle that LV dyssynchrony is part of EA pathology and can be measured from CMR. Our sample size is comparable to that of previously published studies. Only 20% of EA patients in our cohort suffered from severe heart failure. Because all patients were included in a registry, we are optimistic that we could obtain the prospective data in the future.

## Conclusion

EA is characterized by LV intra-ventricular dyssynchrony, which is associated with heart failure and disease severity parameters. CMR derived markers of dyssynchrony can easily be obtained from routine CMR studies and may have a role in the assessment of deterioration of cardiac function, thereby carrying potential value for clinical decision-making.

## Additional files

Additional file 1: Table S1. Comparison of dyssynchrony parameters and torsion/ recoil parameters in a subgroup analysis of EA patients with ASD or previous surgery (“EA ASD/ operation”) vs. EA patients without ASD or previous surgery (“EA no ASD/ no operation”). **Table S2.** Comparison of dyssynchrony parameters and torsion/ recoil parameters in a subgroup analysis of EA patients with ASD or previous surgery (“EA ASD/ operation”) vs. healthy controls (“controls”). **Table S3.** Interobserver analysis of differences in segmentation reproducibility for basal and apical slices. **Table S4.** Correlation of fRV ED and fRV stroke volume (fRV SV) with dyssynchrony parameters (DOCX 18 kb)

## Abbreviations

∅apical: Apical counter-clockwise rotation
∅basal: Basal clockwise rotation
4D: Four dimensional
4D–SDI: 4D volume analysis derived systolic dyssynchrony index
ASD: Atrial septal defect
BNP: Brain natriuretic peptide
CMR: Cardiovascular magnetic resonance
CMR-FT: Cardiovascular magnetic resonance feature tracking
CoV: Coefficient of Variation
C-SDI: Time-to-peak derived circumferential systolic dyssynchrony index
CURE: Circumeferential uniformity ratio estimate
D: Distance
diasTR: Diastolic torsion rate
EA: Ebstein’s Anomaly
ECG: Electrocardiogram
EDVi: End-diastolic volume indexed to body surface area
EF: Ejection fraction
ESVi: Endsystolic volume indexes to body surface area
fRV: Functional right ventricle
FT: Feature tracking
ICC: Interclass Correlation Coefficient
L-SDI: Time-to-peak derived longitudinal systolic dyssynchrony index
LV: Left ventricle/left ventricular
NYHA: New York Heart Association
R/L Index: Total right/ left volume index
R-SDI: Time-to-peak derived radial systolic dyssynchrony index
RURE: Radial uniformity ratio estimate
RV: Right ventricle/right ventricular
RVOT: Right ventricular outflow tract
SDI: Systolic dyssynchrony index
SSFP: Steady state in free precession
SU: Simon Usenbenz
sysTR: Systolic torsion rate
TE: Echo time
TL: Torben Lange
Tor: Torsion
TPK: Time-to-peak
