# Supplementary material for: Left ventricular synchrony, torsion, and recoil mechanics in Ebstein’s anomaly: insights from cardiovascular magnetic resonance
Source: J Cardiovasc Magn Reson. 2017 Dec 14;19:101. doi: 10.1186/s12968-017-0414-y (PMC5729283; doi:10.1186/s12968-017-0414-y)
Supplement: Supplementary file 1 — Comparison of dyssynchrony parameters and torsion/ recoil parameters in a subgroup analysis of EA patients with ASD or previous surgery (“EA ASD/ operation”) vs. EA patients without ASD or previous surgery (“EA no ASD/ no operation”). Table S2. Comparison of dyssynchrony parameters and torsion/ recoil parameters in a subgroup analysis of EA patients with ASD or previous surgery (“EA ASD/ operation”) vs. healthy controls (“controls”). Table S3. Interobserver analysis of differences in segmentation reproducibility for basal and apical slices. Table S4. Correlation of fRV ED and fRV stroke volume (fRV SV) with dyssynchrony parameters (DOCX 18 kb) [file 12968_2017_414_MOESM1_ESM.docx]

|  | **EA ASD/ operation** | **EA no ASD/ no operation** | **p-value** |
| --- | --- | --- | --- |
|  |  |  |  |
| **4D-SDI** | 6,54 ± 2,53 | 7,86 ± 5,20 | 0,825 |
| **C-SDI** | 8,75 ± 4,40 | 7,18 ± 2,83 | 0,386 |
| **RURE** | 0,73 ± 0,038 | 0,71 ± 0,10 | 0,898 |
| **CURE** | 0,77 ± 0,048 | 0,77 ± 0,04 | 0,858 |
| **Torsion** | 1,65 ± 1,34 | 2,32 ± 1,36 | 0,284 |
| **Systolic Torsion Rate** | 11,96 ± 4,13 | 17,38 ± 7,72 | 0,083 |
| **Diastolic Torsion Rate** | -14,72 ± 5,91 | -18,12 ± 7,82 | 0,308 |
| **Max. basal Rotation** | 3,86 ± 1,37 | 6,41 ± 5,14 | 0,665 |
| **Max. apical Rotation** | 4,89 ± 2,69 | 5,83 ± 2,77 | 0,273 |

Table S1: Comparison of dyssynchrony parameters and torsion/ recoil parameters in a subgroup analysis of EA patients with ASD or previous surgery (“EA ASD/ operation”) vs. EA patients without ASD or previous surgery (“EA no ASD/ no operation”)

|  | **EA ASD/ operation** | **Controls** | **p-value** |
| --- | --- | --- | --- |
|  |  |  |  |
| **4D-SDI** | 6,54 ± 2,53 | 2,54 ± 0,62 | **<0,001** |
| **C-SDI** | 8,75 ± 4,40 | 3,80 ± 0,91 | **<0,001** |
| **RURE** | 0,73 ± 0,038 | 0,71 ± 0,09 | **0,004** |
| **CURE** | 0,77 ± 0,048 | 0,77 ± 0,45 | **<0,001** |
| **Torsion** | 1,65 ± 1,34 | 1,76 ± 1,40 | 0,830 |
| **Systolic Torsion Rate** | 11,96 ± 4,13 | 14,31 ± 7,35 | 0,794 |
| **Diastolic Torsion Rate** | -14,72 ± 5,91 | -15,02 ± 9,18 | 0,771 |
| **Max. basal Rotation** | 3,86 ± 1,37 | 4,07 ± 2,24 | 0,408 |
| **Max. apical Rotation** | 4,89 ± 2,69 | 4,41 ± 2,42 | 0,866 |

Table S2: Comparison of dyssynchrony parameters and torsion/ recoil parameters in a subgroup analysis of EA patients with ASD or previous surgery (“EA ASD/ operation”) vs. healthy controls (“controls”)

|  | **ICC** | **CoV** | **Bland-Altmann-Analyis** | |
| --- | --- | --- | --- | --- |
| **Interobserver Analysis** |  |  | |  |
| **Basal Rotation** | 0,92 | 20,90 | | 0,93 (-0,86 – 2,72) |
| **Apical Rotation** | 0,94 | 19,95 | | 0,83 (-1,26 – 2,92) |

Table S3: Interobserver analysis of differences in segmentation reproducibility for basal and apical slices.

|  | **fRV_EF [%]** | **fRV_SVi [ml/m2]** |
| --- | --- | --- |
| **Dyssynchrony Parameter** |  |  |
| **4D-SDI** | **r=-0,462; p=0,013** | r=-0,083 p=0,701 |
| **C-SDI** | **r=-0,395 p=0,028** | r=0,079; p=0,696 |
| **RURE** | r=0,239; p=0,109 | r=0,110; p=0,586 |
| **CURE** | r=0,189; p=0,309 | r=-0,249; p=0,211 |
|  |  |  |

Table S4: Correlation of fRV ED and fRV stroke volume (fRV SV) with dyssynchrony parameters.
